# Adversity History Predicts Self-Reported Autonomic Reactivity and Mental Health in US Residents During the COVID-19 Pandemic

**DOI:** 10.3389/fpsyt.2020.577728

**Published:** 2020-10-27

**Authors:** Jacek Kolacz, Lourdes P. Dale, Evan J. Nix, Olivia K. Roath, Gregory F. Lewis, Stephen W. Porges

**Affiliations:** ^1^Traumatic Stress Research Consortium, Kinsey Institute, Indiana University, Bloomington, IN, United States; ^2^Department of Psychiatry, College of Medicine-Jacksonville, University of Florida, Jacksonville, FL, United States; ^3^Intelligent Systems Engineering, Indiana University, Bloomington, IN, United States; ^4^Department of Psychiatry, University of North Carolina at Chapel Hill, Chapel Hill, NC, United States

**Keywords:** coronavirus, COVID-19, autonomic nervous system, polyvagal theory, PTSD, depression, worry, trauma

## Abstract

**Background:** The spread of the COVID-19 virus presents an unprecedented event that rapidly introduced widespread life threat, economic destabilization, and social isolation. The human nervous system is tuned to detect safety and danger, integrating body and brain responses via the autonomic nervous system. Shifts in brain-body states toward danger responses can compromise mental health. For those who have experienced prior potentially traumatic events, the autonomic threat response system may be sensitive to new dangers and these threat responses may mediate the association between prior adversity and current mental health.

**Method:** The present study collected survey data from adult U.S. residents (*n* = 1,666; 68% female; Age *M* = 46.24, *SD* = 15.14) recruited through websites, mailing lists, social media, and demographically-targeted sampling collected between March and May 2020. Participants reported on their adversity history, subjective experiences of autonomic reactivity, PTSD and depression symptoms, and intensity of worry related to the COVID-19 pandemic using a combination of standardized questionnaires and questions developed for the study. Formal mediation testing was conducted using path analysis and structural equation modeling.

**Results:** Respondents with prior adversities reported higher levels of destabilized autonomic reactivity, PTSD and depression symptoms, and worry related to COVID-19. Autonomic reactivity mediated the relation between adversity and all mental health variables (standardized indirect effect range for unadjusted models: 0.212–0.340; covariate-adjusted model: 0.183–0.301).

**Discussion:** The data highlight the important role of autonomic regulation as an intervening variable in mediating the impact of adversity on mental health. Because of the important role that autonomic function plays in the expression of mental health vulnerability, brain-body oriented therapies that promote threat response reduction should be investigated as possible therapeutic targets.

## Introduction

On January 21, 2020, the Centers for Disease Control and Prevention announced the first confirmed case of the 2019 Novel Coronavirus (2019-nCoV) in the United States (1). Following this initial case, the virus spread rapidly throughout the country. Due to the drastic global spread of the virus the World Health Organization labeled the outbreak as a pandemic on March 11, 2020 and a national emergency was declared in the U.S. on March 13 (2, 3). The spread of the COVID-19 virus is an unprecedented event that rapidly introduced the threat of loss of life, severe illness, unemployment, economic destabilization, and social isolation. The danger, uncertainty, and social seclusion all have a potential to jeopardize well-being and mental health, with preliminary data and projections pointing to elevated rates of mental illness and distress (4, 5). Research, health policy, and intervention implementation all require information on factors that impact mental health vulnerability and resilience during this time (6) as well as identifying mechanisms through which mental health is challenged.

The human nervous system is tuned to detect safety and danger, integrating the body and brain through the autonomic nervous system [ANS, (7–9)]. The ANS is crucial for coordinating brain and body functions in safe contexts and promoting defensive bio-behavioral reactions during threat (10–14). The ANS forms efferent (motor) and afferent (sensory) connections that are integrated throughout the brainstem, spinal cord, and body organs. These circuits promote maintenance and reactivity in a range of physical functions such as cardiac output, sweating, breathing, and digestion. Shifts in physiological states toward danger-responses inhibit safety-related functions throughout the body. These shifts, particularly in the long term, can compromise emotional and physical health, influencing cognitive and emotional processes through pathways that connect higher level brain regions with the brainstem (15, 16).

The polyvagal theory describes how the structural and functional organization of human threat response systems are rooted in phylogenetic heritage (7, 8, 16, 17). The emergence of mammals was marked by the integration of ANS pathways with circuits that regulate social communication, forming a neuroanatomical social engagement system that dampens defense responses via the ventral vagal pathway of the parasympathetic nervous system and promotes affiliative social interactions. As proposed by the theory, danger detection can trigger withdrawal of the social engagement system, which can promote responses that include mobilization and immobilization (e.g., shut down). Mobilization states, in the absence of the active social engagement that down-regulates defenses, provide a neural platform for fight and flight behaviors. These mobilization states can contribute to chronic anxiety or irritability. Immobilization states, in turn, provide a platform for withdrawal and depression. Both defensive strategies have adaptive value for protecting the individual from certain types of threat, but interfere with co-regulation and feelings of safety.

Dampened parasympathetic activity is associated with depression, anxiety, and post-traumatic stress disorders (18–21). Although there are many external influences on the ANS, the most direct evidence supporting a causal association between the ANS and mental health come from vagal nerve stimulation (VNS), which uses an electrical current to stimulate vagal afferent pathways that lead from the periphery to the brainstem. There is now substantial evidence that vagal nerve stimulation can improve depression symptoms (22, 23) and modulate anxiety and fear (24, 25), supporting a causal connection between autonomic signaling and emotional well-being.

Prior experience with adversity may sensitize a nervous system toward more pronounced mental and physical health changes in response to danger (15, 26–28). In humans the ANS is developmentally sensitive to safety and threat cues and highly responsive to environmental conditions (29–31). This sensitivity promotes a potential mechanism for how adverse experiences may re-tune nervous systems and alter threat responses to future dangers. Thus, the individual's autonomic state might function as an intervening variable determining whether cues of threat are buffered or function as potent disruptors.

Children and adults with a history of childhood maltreatment are more likely to have blunted parasympathetic activity (32, 33). Dampened parasympathetic activity is associated with post-traumatic stress disorders (19), and can be seen in adults with a maltreatment history even when they do not meet clinical diagnostic criteria for PTSD (27, 32). Evidence for a causal pathway between child maltreatment and autonomic regulation has been demonstrated through randomized intervention studies. In one study, children living in Romanian orphanages with access to physical needs but lacking in emotional caregiver connection exhibited low parasympathetic activity and less flexibility in response to challenges, but those who were randomized into foster care that provided greater emotional interpersonal connection developed autonomic activity much like their peers who had never been institutionalized (34). In a recent study of children referred to Child Protective Services for maltreatment, children developed better parasympathetic flexibility in response to challenges when their caregivers were randomized into a parenting sensitivity intervention compared to peers in a control condition (35).

Taken together, theory and empirical evidence reviewed above supports the possibility that prior adversity could help shape autonomic reactivity in response to threats, which may increase worry in response to danger, and the risk of developing PTSD and depressive symptomology. Those with a prior adversity history are at risk for higher threat-response autonomic activity at rest and stronger responses to threatening challenges (36–38). Recent longitudinal data from a cohort study show that stressful life events measured prior to the pandemic are predictive of emotional distress in young adults (39). The COVID-19 combination of life threat, economic destabilization, and social isolation create a particularly challenging environment for the nervous system, placing individuals at risk of mental and physical problems and exacerbation of pre-existing conditions (40).

Using a combination of social media recruitment and targeted online panels data collection, this cross-sectional survey study sought to examine the relations between prior adversity, autonomic reactivity, mental health, and concerns about the coronavirus during the first months of the pandemic among U. S. residents (March–May 2020). The specific aim was to examine whether self-reported autonomic reactivity mediates the relationship between prior adversity and current depression/PTSD symptomatology and worry during the COVID-19 pandemic. Based on prior literature, we hypothesized that self-reported autonomic reactivity would be related to previous adversity, current mental health, and worry about COVID-19, and that it would be mediate the relationship between these variables.

## Materials and Methods

### Procedure

The protocol was approved by Indiana University's Institutional Review Board. All participants provided informed consent for the study. Data collection was conducted online from March 29 to May 13, 2020. The study recruited from a general population with inclusion criteria being that participants must be 18 years or older. Recruitment was conducted via social media postings on Twitter, Facebook, Instagram, Reddit, and email lists. Additional recruitment oversampling for male, low income, and non-Caucasian responders in the U.S. was conducted via Qualtrics Panels. Qualtrics Panels consist of respondents who have signed up to participate in online surveys in exchange for incentives including cash, airline miles, and gift cards and can be targeted by demographic categories. Participants who completed the survey through Qualtrics Panels were paid according to their compensation agreement with the service. Paid commercial online panel data has been found to have similar scale internal reliability estimates and effect sizes between variables compared to conventional sampling techniques (41). In the United States, samples recruited by Qualtrics are most demographically similar to a national probability sample compared to other online sampling services (42).

The study landing page, which was linked directly from recruitment advertisements, was accessed 5,240 times. Of these, 3,817 individuals consented to participate. Data quality analysis was conducted by automated checks for poor quality responses and manual inspection. Responses with large sections of identical responses for any one survey section were flagged and checked for plausibility, internal consistency, comparison to item response patterns in prior studies. Responses that did not meet these requirements or had a completion time faster than 25% of the median completion time were excluded.

### Measures

#### Previous Adversity

The Adverse and Traumatic Experiences Scale (43) was created to inquire about a range of adverse and traumatic experiences that had been included in other measures including the ACES (44), Trauma History Questionnaire (45), Life Events Checklist for DSM-5 (46), and Brief Trauma Questionnaire (47). Thus, the measure asks about childhood adverse experiences, childhood maltreatment, other person maltreatment, life-threatening situations, sudden deaths of close ones, and personal health situations. To test study hypotheses, adverse experiences relating to physical health were excluded because of the elevated risk of serious illness due to COVID in those with prior medical conditions. Thus, respondent-reported prior adverse events of maltreatment, life-threatening situations, and sudden deaths of close ones were summed to create an adversity score (range: 0–19).

#### Self-Reported Autonomic Reactivity

The Body Perception Questionnaire Short Form [BPQ-SF; (48, 49)] was used to measure self-reported experiences of reactivity in organs and tissues that are regulated by the autonomic nervous system. The BPQ-SF has been found to have good psychometric properties, convergent validity with similar measures, and consistent factor structure across samples [(50); Kolacz et al., in preparation; Cerritelli et al., under review]. The combined autonomic reactivity subscale assesses the typical experience of the reactivity of functions above the diaphragm (e.g., sweat in armpits) and gastrointestinal functions (e.g., constipation, indigestion) on a 5-point Liker-type scale (ranging from “never” to “always”). Raw scores were transformed into T scores based on previously collected norms (49). Higher scores on the subscale are indicative of destabilized autonomic reactivity and associated with lower parasympathetic activity, higher resting heart rate, and less parasympathetic and sympathetic flexibility in response to a challenge (Kolacz et al., in preparation).

#### Post-traumatic Stress Disorder Symptoms

PTSD symptoms were measured using the PTSD Checklist-Civilian Version (51), a 17-item self-report measure assessing level of re-experiencing, avoidance, and hyperarousal related to experiencing a traumatic event. It has been found to have good internal stability, test-retest reliability, convergent validity, and temporal stability (52). The items were developed to correspond to DSM-IV-TR criteria for PTSD (53) and measure problems in response to stressful life experiences over the past month using a five-point Likert-type scale (0 = not all, 1 = a little bit, 2 = moderately, 3 = quite a bit, 4 = extremely). On the PCL-C, endorsement of at least one re-experiencing item, at least three avoidance items, and at least two hyperarousal items is suggestive of symptoms that may meet PTSD diagnosis (54).

#### Depression Symptoms

The Patient Health Questionnaire-2 was used as a depression screener (55, 56). The instrument inquires about frequency of depressed mood and anhedonia over the past 2 weeks using a 4-point Likert-type scale (0 = not at all, 1 = several days, 2 = more than half the days, and 3 = nearly every day). The scores for the two items are summed to determine a total score, with a score of 3 or greater suggesting that the individual should be assessed further to determine whether depressive disorder criteria is met.

#### COVID-19-Related Worry

Respondents reported on their extent of worry about becoming infected with the COVID-19 virus, seriously ill due to the virus, unable to access important necessities such as a food and medication, unemployed (i.e., losing their jobs), and less financially stable. For each item, the participants reported their level of worry via a 4-point Likert-type scale (0 = not worried, 1 = a little worried, 2 = somewhat worried, and 3 = very worried).

### Data Analysis

Analysis was conducted in R 3.6.2 (57). Continuous variables were examined for group differences using Welch's unequal variances *t*-test, a more robust alternative to Student's *t*-test for groups that may have unequal variances or sample sizes (58); categorical variables were examined with χ2 tests; and ordinal variables with Mann-Whitney-Wilcoxon tests. Cohen's d was used to determine standardized mean difference, a measure of effect size of differences between groups.

Formal mediation analysis was conducted using path analysis and structural equation modeling using the Lavaan package (59). Mediation models are statistical tests that assess whether the association between an independent and dependent variable can be attributed to the effect of a third variable (60–62). In mediation analysis, the strength of mediation is represented via the indirect effect (the product of the coefficient of the independent variable on the mediator and the mediator on the outcome variable). The direct effect is the association of the independent variable on the dependent variable, adjusting for the effect of the hypothesized mediator. The total effect is the sum of the direct and indirect effects.

Models were estimated using diagonally-weighted least squares. The full weight matrix was used to compute robust standard errors, and the test statistic was mean- and variance-adjusted. Indirect and total effect confidence intervals were calculated using bias-corrected adjusted bootstrap percentiles with 5,000 draws. Compared to other mediation estimation methods, this method has been found to have superior power for detecting true effects with accurate Type I error rates (63). Mediation was supported if the bootstrapped 95% confidence interval around the indirect effect did not include 0. Total effects were examined for evidence of divergence of direction between direct and indirect effects, which may weaken, nullify, or reverse the indirect effect. Binary endogenous variables were modeled using probit link functions. Age and gender were included as exogenous variables to adjust model estimates.

Model fit was evaluated using the root mean squared error of approximation [RMSEA; (64)], the Tucker-Lewis Index [TLI; (65)]; and the Comparative Fit Index [CFI; (66)]. Based on recommendations from Hu and Bentler (67), good model fit was evidenced by RMSEA values near or below 0.06 as well as CFI and TLI values near or above 0.95. When model fit was poor, modification indices were cautiously examined to determine whether freeing certain parameters would improve model fit. Modification indices provide data-driven information on the amount that model fit would improve if a single parameter restriction were lifted from the model. Given that modification indices are susceptible to capitalizing on chance characteristics of the data (68), decisions based on modification indices were used sparingly and applied only when the resulting model change could be supported by theory.

## Results

The final sample size, excluding incomplete responses (*n* = 995), poor-quality data (*n* = 303 from the paid panel recruitment, *n* = 2 from the social media recruitment), and demographic criteria (e.g., non-US citizen; *n* = 851) was 1,666 (See Supplementary Materials for a detailed consort diagram). Demographic variable descriptive statistics are reported in Table 1. Survey respondents ranged from 18 to 88 years of age (M = 45.87; SD = 16.17) and were slightly oversampled with regard to females, high yearly household income (>50% reporting $60 k or more), and higher levels of education (44.5% holding a graduate degree).

**Table 1:** Sample descriptive statistics.

**Variable**	**Descriptive statistics**
*n*	1,666
Age (M ± SD)	45.87 ± 16.17
Age range (years)	18–88
**Gender**	
Female	994 (59.7%)
Male	647 (38.8%)
Non-binary	13 (0.8%)
Transgender	1 (<0.1%)
Unspecified	11 (0.7%)
**Race and/or Ethnicity**	
White or Caucasian	1,175 (70.5%)
Black or African American	163 (9.8%)
Hispanic/Latino(a)	106 (6.4%)
Asian/Pacific Islander	94 (5.6%)
American Indian or Alaska Native	12 (0.7%)
Native Hawaiian or Other Pacific Islander	3 (0.2%)
Additional Races and/or Ethnicities (self–described)	6 (0.4%)
Multiracial	88 (5.3%)
Unspecified	19 (1.1%)
**Income (USD)**	
< $20,000	233 (14.1%)
$20,001–$60,000	562 (33.7%)
$60,001–$100,000	410 (24.6%)
> $100,001	449 (27.2%)
Unspecified	12 (0.7%)
**Education level**	
Graduate degree	741 (44.5%)
College or University	620 (37.3%)
Secondary school/High school	282 (16.9%)
Primary school	16 (1.0%)
Vocational school	5 (0.3%)
Unspecified	2 (0.1%)

Autonomic reactivity T scores had similar distributional features to those reported in previous studies (M = 48.07, SD = 10.15, Range: 33.23–83.45) (49, 50). The mean number of prior adverse events was 5.93 (SD = 4.86; range: 0–19). Respondents reported high levels of worry about the negative effects of COVID-19, with the highest levels of worry relating to infection, serious illness caused by the virus, and loss of financial stability (Table 2). Of the respondents, 93.7% reported at least a little worry about 1 or more threats associated with the virus, 27.8% met symptom criteria for post-traumatic stress disorder, and 28.7% met symptom criteria for depression.

**Table 2:** COVID-19-related worry response distributions.

**COVID-19 worry variable**	**Not worried (%)**	**A little worried (%)**	**Somewhat worried (%)**	**Very worried (%)**
Becoming infected with COVID-19 virus	17.54	35.80	29.55	17.12
Becoming seriously ill because of coronavirus	22.56	34.72	25.87	16.85
Being unable to get important necessities	36.93	26.75	21.87	14.46
Being unable to get necessary medications	44.80	24.26	18.20	12.73
Losing job	49.12	18.47	14.48	17.92
Becoming less financially stable	23.00	26.49	23.42	27.09

Pearson and point-biserial correlations for continuous and binary variables are presented in Table 3. Age had a very small negative association with number of prior adversities (*r* = −0.07). In addition, age had a negative relation with self-reported autonomic reactivity (*r* = −0.23) and more advanced age was associated with a lower probability of depression and PTSD symptoms (*r* = −0.26 and −0.27, respectively). Higher numbers of previous adverse events were associated with higher values of autonomic reactivity (*r* = 0.60, *p* < 0.0001). Respondents with depression symptoms had more prior adverse experiences (No symptoms M = 5.07, SD = 4.10; Symptoms M = 8.10, SD = 5.84; *t*_(664.49)_ = 10.317, *p* < 0.0001, Cohen's d = 0.65). Those who had PTSD symptoms likewise had more prior adverse experiences (No symptoms M = 4.72, SD = 3.67; Symptoms M = 9.07, SD = 6.17; *t*_(590.11)_ = 14.465, *p* < 0.0001, Cohen's d = 0.98). Respondents who met depression criteria reported more destabilized autonomic reactivity [No symptoms M = 45.18, SD = 8.99; Symptoms M = 55.28, SD = 12.37, *t*_(686.98)_ = 16.201, *p* < 0.0001, Cohen's d = 1.00]. Likewise, destabilized autonomic reactivity was also greater in those who met PTSD criteria (No symptoms M = 44.56, SD = 8.05; Symptoms M = 57.13, SD = 12.45, *t*_(616.82)_ = 20.17, *p* < 0.0001, Cohen's d = 1.33). Household income was not associated with number of adverse experiences (rho = 0.01, *p* = 0.77) or autonomic reactivity (rho = −0.05, *p* = 0.05). There were significant but small negative associations of household income and education with depression (rho = −0.10, *p* < 0.001; rho = −0.05, *p* = 0.03, respectively) and PTSD symptoms (rho = −0.10, *p* < 0.001; rho = −0.07, *p* = 0.01, respectively).

**Table 3:** Means, standard deviations, ranges, and correlations with confidence intervals.

**Variable**	**1**	**2**	**3**	**4**
1. Age				
2. Prior adversities	−0.07^**^			
	[−0.12, −0.02]			
3. Autonomic reactivity	−0.23^**^	0.60^**^		
	[−0.27, −0.18]	[0.57, 0.63]		
4. Depression symptoms (Binary)	−0.26^**^	0.28^**^	0.41^**^	
	[−0.31, −0.22]	[0.24,0.33]	[0.37, 0.45]	
5. PTSD symptoms (Binary)	−0.27^**^	0.40^**^	0.51^**^	0.54^**^
	[−0.31, −0.22]	[0.36, 0.44]	[0.47, 0.55]	[0.50, 0.57]

*Values in square brackets indicate the 95% confidence interval for each correlation coefficient*.

*p < 0.05.

** *p < 0.01*.

*Correlations between continuous variables are calculated using Pearson's correlation coefficient and those including binary variables are point-biserial correlations*.

Younger respondents expressed more worry about loss of access to necessities, loss of access to medication, loss of job, and loss of financial stability due to the coronavirus (age and worry item rho = −0.21, −0.18, −0.30, −0.24, respectively; all *p* < 0.001) but there were no associations of age with worry about contracting the virus or becoming seriously ill because of it (rho = −0.02, *p* = 0.39; rho = 0.03, *p* = 0.29; respectively).

### COVID-Related Worry Measurement Model

**Figure 1:**
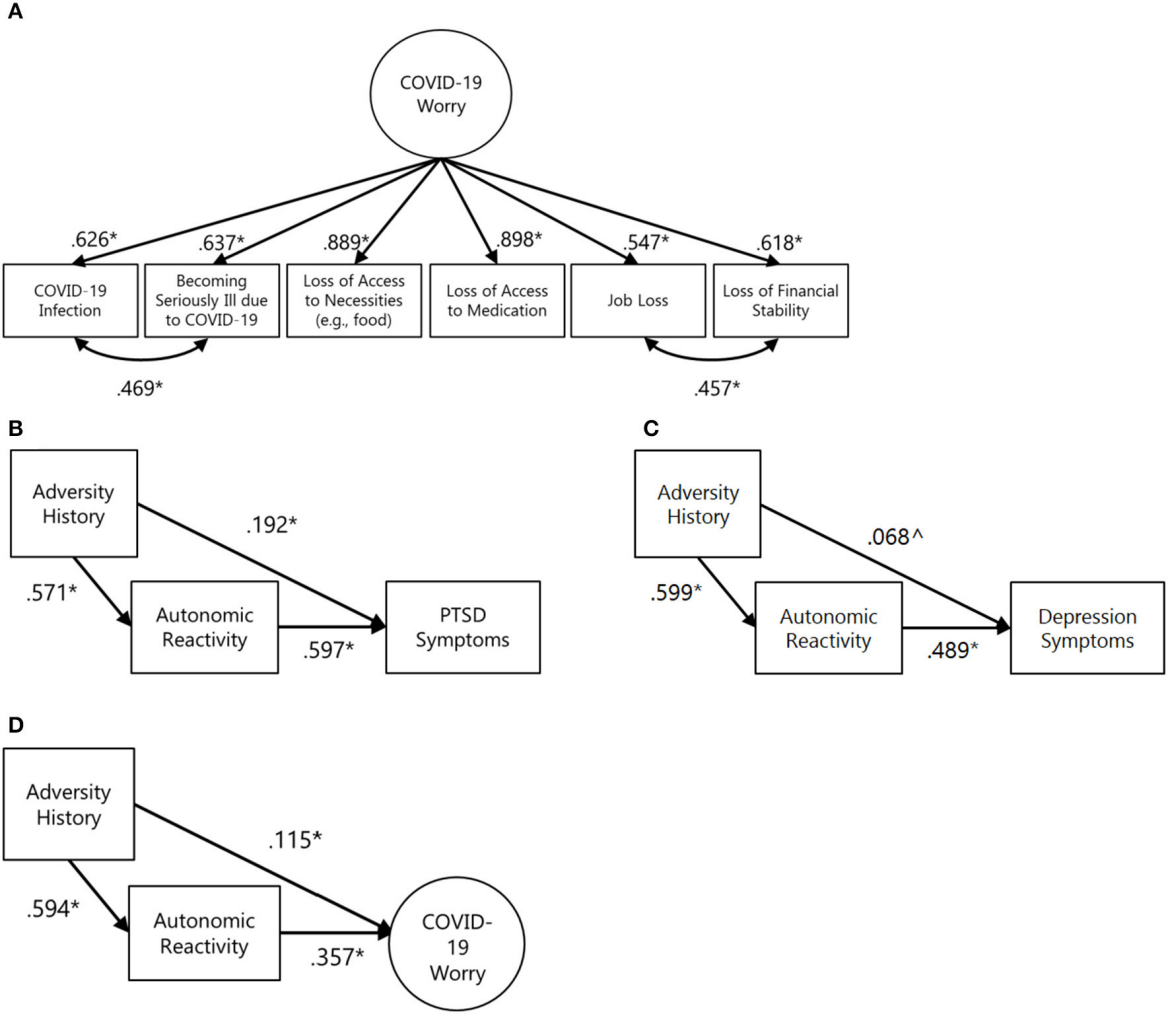
COVID-19 worry measurement model and preliminary, unadjusted mediation models for individual predictors. In all figures: *p < 0.05, ^∧^*p* < 0.10. **(A)** COVID-19 worry measurement model. Model fit indices: χ2 = 12.264, df = 7, RMSEA = 0.021 [90% CI: 0.000, 0.041] CFI = 1.000 TLI = 1.000 **(B)** Unadjusted mediation test for PTSD symptom outcome with standardized coefficients. Model fit indices not available due to model saturation. Standardized indirect effect = 0.340 [95% CI: 0.291, 0.390], standardized total effect = 0.532 [95% CI: 0.462, 0.602]. **(C)** Unadjusted mediation test for depression symptom outcome with standardized coefficients. Model fit indices not available due to model saturation. Standardized indirect effect = 0.293 [95% CI: 0.244, 0.342], standardized total effect = 0.361 [95% CI: 0.296, 0.429]. **(D)** Unadjusted mediation test for COVID-related worry outcomes with standardized coefficients. χ2 = 27.045, df = 17, RMSEA = 0.019 [90% CI: 0.000, 0.032] CFI = 1.000 TLI = 1.000. Standardized indirect effect = 0.212 [95% CI: 0.160, 0.266], standardized total effect = 0.327 [95% CI: 0.264, 0.385].

Modeling began with establishing a COVID-19 worry using a reflective measurement model. In this model, each worry indicator has a unique influence independent of others and is also influenced by a general worry latent factor (69). Modification indices supported the need for free co-variances between (a) worry about infection and worry about becoming seriously ill and (b) worry about losing one's job and becoming less financially stable (i.e., these paths were not constrained to 0). These appeared to reflect the added correlation of infection-related and income-related worries and could thus be justified as modifications to the model. When these covariances were included, the measurement model fit the data well (χ2 = 12.264, df = 7, CFI = 1.000, TLI = 1.000; Figure 1A).

### Model Building

Modeling proceeded by conducting individual tests of mediation for each outcome variable–PTSD symptoms, depression symptoms, and COVID-19-related worry.

First, joint variable distributions of adversity history, self-reported autonomic reactivity, and PTSD symptoms were examined. Formal testing supported the mediation of autonomic reactivity between adversity history and PTSD symptoms (standardized indirect effect = 0.340 [95% CI: 0.291, 0.390], standardized total effect = 0.532 [95% CI: 0.462, 0.602]; Figure 1B). Second, key variable relations with depression symptoms were examined. Formal testing supported the mediation of autonomic reactivity between adversity history and depression symptoms (standardized indirect effect = 0.293 [95% CI: 0.244, 0.342], standardized total effect = 0.361 [95% CI: 0.296, 0.429]; Figure 1C). Third, key variable relations with COVID-related worry was examined. The extent of worry was positively associated with autonomic reactivity (*r* = 0.357). As above, formal testing supported the mediation of autonomic reactivity between adversity history and COVID-related worry (standardized indirect effect = 0.212 [95% CI: 0.160, 0.266], standardized total effect = 0.327 [95% CI: 0.264, 0.385]; Figure 1D).

**Figure 2:**
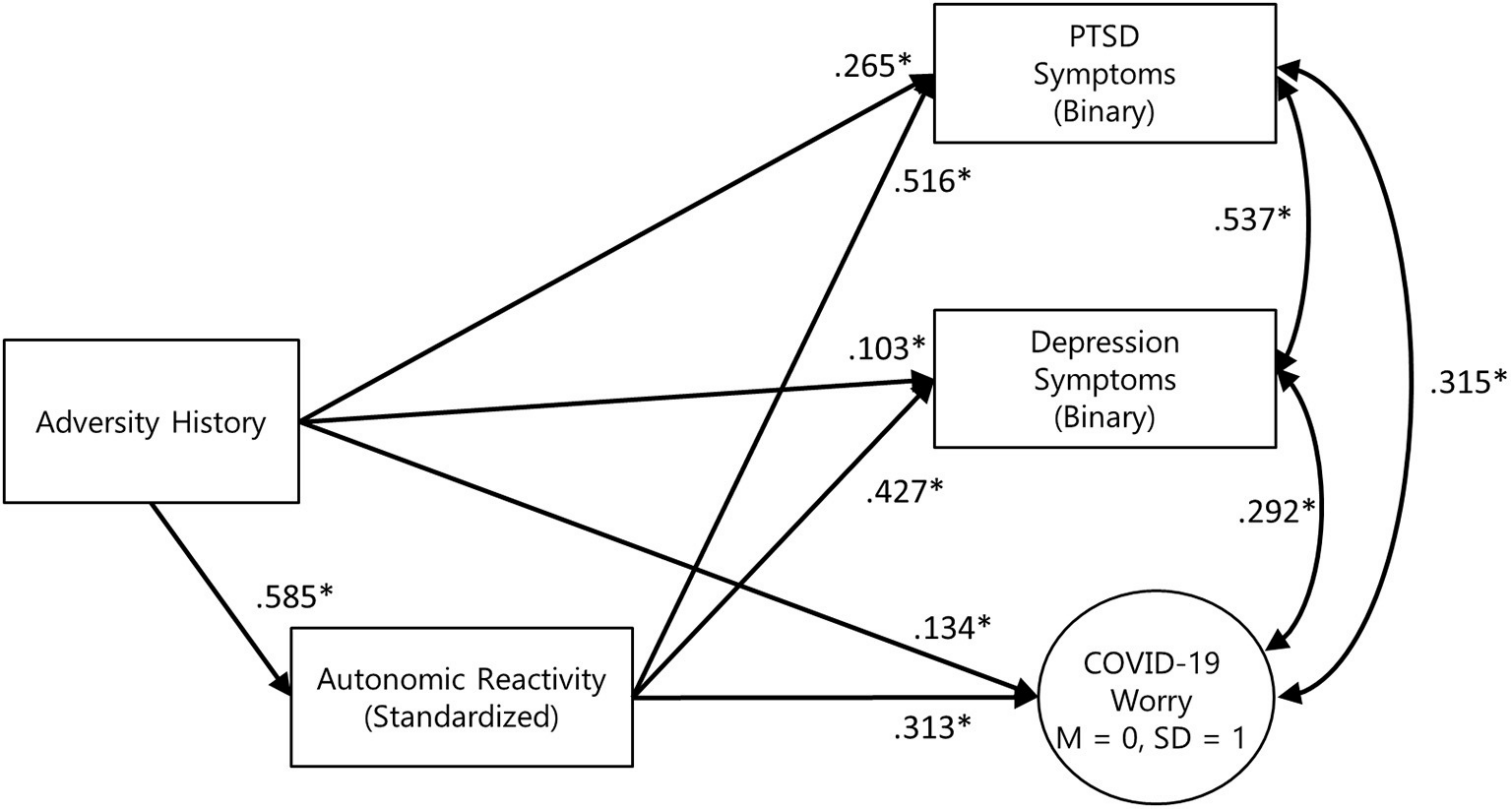
Simplified final model diagram of the 3-outcome mediation model, adjusted for age and sex. Model fit indices: χ2 = 216.853, df = 37, RMSEA = 0.056 [90% CI: 0.049, 0.063] CFI = 0.994 TLI = 0.994. PTSD symptom standardized indirect effect = 0.301 [95% CI: 0.251, 0.354], standardized total effect = 0.558 [95% CI: 0.476, 0.631]. Depression symptom standardized indirect effect = 0.250 [95% CI: 0.202, 0.303], standardized total effect = 0.353 [95% CI: 0.283, 0.423]. COVID-19 worry standardized indirect effect = 0.183 [95% CI: 0.130, 0.237], standardized total effect = 0.318 [95% CI: 0.255, 0.383].

**Figure 3:**
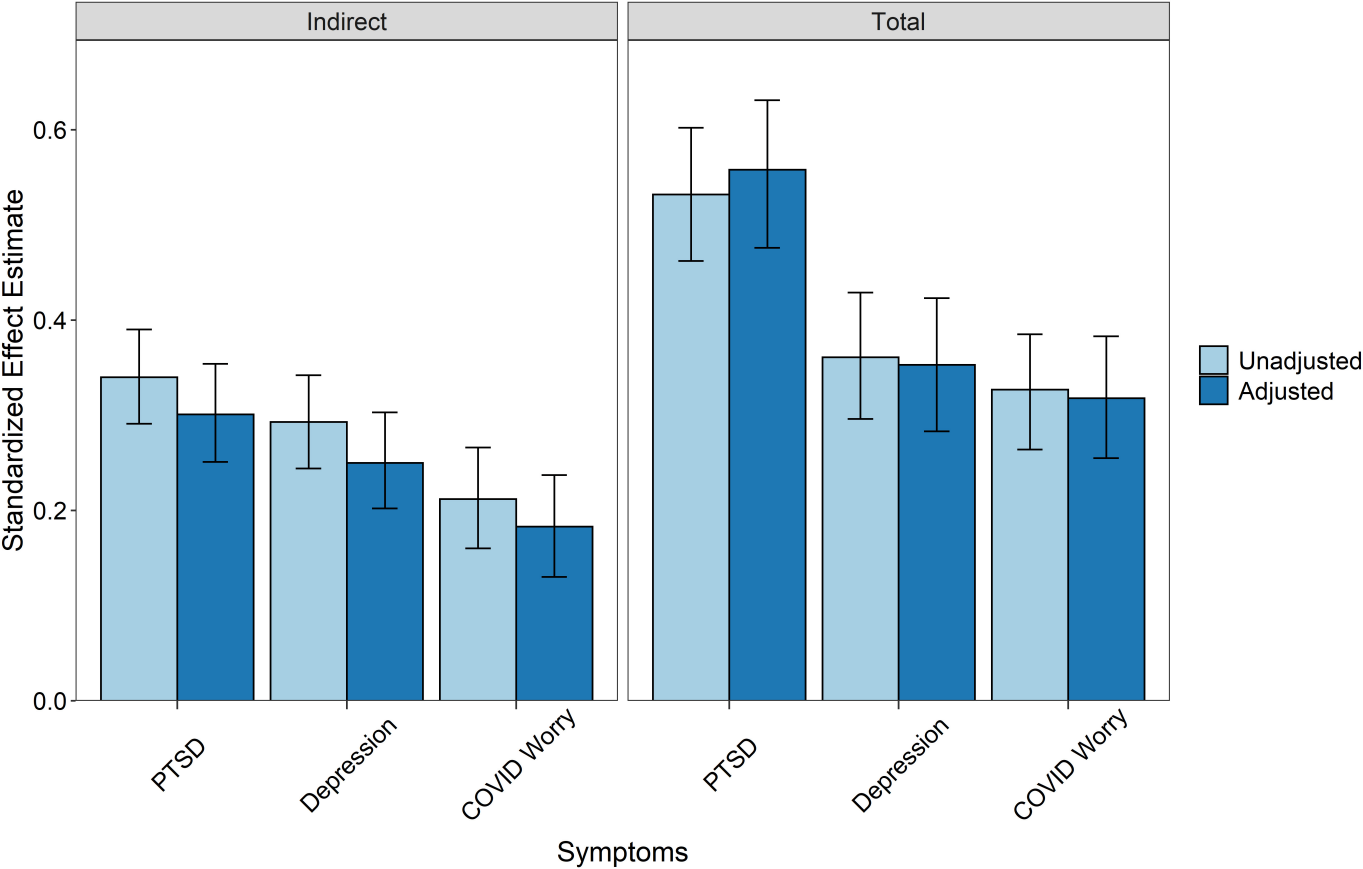
Indirect and total effects from mediation models with 95% confidence intervals. Indirect effects represent the mediation strength of the adversity -> self-reported autonomic reactivity -> outcome pathway. The total effects are the sum of direct and indirect effects in the models. Unadjusted models are calculated using the paths between the three key variables only. Unadjusted estimates for the PTSD model: Standardized indirect effect = 0.340 [95% CI: 0.291, 0.390], standardized total effect = 0.532 [95% CI: 0.462, 0.602]. Unadjusted estimates for the depression model: Standardized indirect effect = 0.293 [95% CI: 0.244, 0.342], standardized total effect = 0.361 [95% CI: 0.296, 0.429]. Unadjusted estimates for the COVID-19 worry model: Standardized indirect effect = 0.212 [95% CI: 0.160, 0.266], standardized total effect = 0.327 [95% CI: 0.264, 0.385]. Adjusted models include all outcome variables with gender and age covariates. PTSD symptom standardized indirect effect = 0.301 [95% CI: 0.251, 0.354], standardized total effect = 0.558 [95% CI: 0.476, 0.631]. Depression symptom standardized indirect effect = 0.250 [95% CI: 0.202, 0.303], standardized total effect = 0.353 [95% CI: 0.283, 0.423]. COVID-19 worry standardized indirect effect = 0.183 [95% CI: 0.130, 0.237], standardized total effect = 0.318 [95% CI: 0.255, 0.383].

The three mediation models were combined to test the independence of effects, with gender and age included as exogenous predictors of adversity history, autonomic reactivity, and all outcome variables. Due to small numbers of respondents who identified as non-binary or transgender (*n* = 14), only male and female effects could be included in the model. Model results are presented in Figure 2. All outcome variables were positively correlated, with the strongest association being between PTSD and depression symptoms (*r* = 0.537). Adjusting for age, gender, and the mutual associations between outcome variables, formal testing supported the mediation of autonomic reactivity in the link between adversity history and PTSD symptoms, depression symptoms, and COVID-19-related worry (Figure 3). Inclusion of household income in sensitivity analyses did not substantively affect the pattern of results.

## Discussion

This cross-sectional survey study focused on a large general sample of US residents and the factors that may influence patterns of mental health in response to the coronavirus pandemic. It examined the potential impact of adversity (i.e., childhood adversity/maltreatment, intimate partner maltreatment, life-threatening events, and sudden losses) and self-reported autonomic reactivity. The results support the hypothesis that self-reported autonomic reactivity was related to previous adversity and current mental health. Destabilized autonomic reactivity scores were higher in respondents that reported experiencing more prior adverse events, and those who met the symptom criteria for depression and/or PTSD.

This study suggests that prior adversity history is a risk factor for mental health and worry during the COVID-19 pandemic, and that these effects are mediated by autonomic dysregulation. To our knowledge, this is the first study to examine the link between adversity history, autonomic reactivity, and a large-scale external stressor such as a pandemic. Adjusting for age, gender, and the mutual associations between outcome variables, formal testing supported the mediation of autonomic reactivity in the link between prior adversity and PTSD symptoms, depression symptoms, and COVID-19-related worry. These findings are consistent with Polyvagal Theory and previous research suggesting that individuals who experience adversity are at increased risk of developing chronic and sensitized threat responses to new challenges (27, 32). They are also consistent with research suggesting that autonomic dysregulation is a linking component that is found in a range of clinical conditions including anxiety (70), disorders of impulse control (71), borderline personality disorder (72), and PTSD (73). The mechanism in this study may also be related to neuroticism, a relatively stable tendency to respond to events with negative emotions and lability, which appears to increase risk of mental health disorders (74). Sensitized or chronic autonomic threat reactions may influence long term patterns of emotional responses toward negativity. There is some evidence of dampened parasympathetic regulation and sensitized physiological reactivity in those who fit a neurotic profile (75, 76) though this connection has been understudied in the context of adversity history and responses to prolonged external danger.

Although the current study does not focus on a clinical sample, the result suggesting that autonomic reactivity may be a mechanism linking adversity and psychological function may have implications for mental health intervention and prevention strategies. These results point to the brain-body threat-response circuits that impact physical, emotional, and cognitive function, suggesting that improving their regulation during a crisis may be a promising target for improving mental health and worry. Thus, it may be beneficial for research to examine how therapeutic strategies for dampening chronic threat responses and improving safety-related regulation as part of trauma interventions can help individuals whose nervous systems are biased toward mobilization and/or shut down. These safety-focused strategies could help with the stabilization that is needed prior to attempting other approaches, especially those involving exposure therapy.

Our results are consistent with clinical insights that individuals experiencing mental health symptoms may benefit from interventions with bottom-up approaches focused on the affect and feelings within the body [i.e., body-based or sensorimotor; (77)]. These approaches (e.g., sensorimotor psychotherapy and relaxation training) use interoception techniques (i.e., the noting of sensations, discomforts, pain, tension, pleasurers, and cues) to increase positive feelings toward physical sensations and help with integrating sensations and body regulation (78). Interventions including yoga (79), mindfulness-based stress reduction (80), and biofeedback (81) have been shown to reduce threat-responsive autonomic reactivity and have benefits for mental health. Additional research should explore their use as a therapeutic method or as part of a multi-method intervention to assist with coping during large scale crises such as the COVID-19 pandemic.

In addition, laboratory studies have shown that social connections can inhibit threat responses and promote affiliative safety states (82, 83). The social distancing and isolation strategies put in place by government mandates and individual decisions to reduce the spread for the COVID-19 virus may be detrimental if they decrease opportunities for co-regulation with others to reduce the impacts of threat response reactivity. This suggests that research into the promotion of opportunities for socioemotional connections during times of physical distancing is an important target to improve understanding of how clinicians can support coping mechanisms and help clients regulate threat responses.

### Limitations

This study is not without limitations, including the use of social media for online data collection. To reduce the data bias, procedures were followed to evaluate data quality using a combination of attention checks and statistically or logically implausible response patterns. Given the social media sampling strategy, this study was not designed to assess nationally representative prevalence rates, though the relations between variables are consistent with prior literature based on objective measures, experimental methods, and prospective designs. The strength of the cross-sectional design selected for this study is the ability to rapidly collect data using validated measures to provide a picture of responses during the first months of the pandemic.

Another limitation relates to the use of self-report measures. Retrospective reporting of prior adverse events may induce bias both toward over- or under-reporting, which can contribute to decreasing reliability and validity of measurement (84) and bias the associations of self-reports compared to objective reports (85). However, the strengths of adversity self-reports include sensitivity for events that may not have been captured by prospective measures, such as the low documentation of sexual abuse in official records (85). In addition, the psychometric properties of the COVID-19 worry measure have not yet been examined in other datasets. The measure was created by a team of researchers and clinicians to address pressing needs at a time when no validated measure was available. The measurement model described in this study provides a starting point for additional psychometric study in the future. Follow up studies will need to examine test-retest reliability, validity, and whether the factor structure of worry is consistent across samples.

Further, due to single time point design it is unknown if the participants were already experiencing symptoms of PTSD, depression, and economic worry prior to the pandemic. Objective autonomic monitoring and prospective longitudinal designs are needed to support the findings reported here, and to better establish temporal precedence. However, the results presented here are consistent with longitudinal data that show adverse experiences reported prior to the onset of the pandemic are a predictor of emotional distress (39). Thus, there is a need for prospective longitudinal research that allows for a better understanding about how changes in mental health relate to autonomic reactivity and regulation. Future research should also address the contributions of cognitive processes, such as posttraumatic growth and worldview, which are affected by adversity.

## Conclusion

The autonomic nervous system (ANS) integrates brain-body threat responses. Prior adversity may sensitize individuals toward autonomic threat responses that increase risk of mental health and worry during crises such as the COVID-19 pandemic. In light of prior literature that shows the ANS to be sensitive to context and a useful therapeutic target, the results support the need for research on whether reduction of bio-behavioral threat responses and improvement of safety-related autonomic function could be effective treatment strategies, particularly during chronic, uncontrollable stressors.

## Data Availability Statement

The raw data supporting the conclusions of this article will be made available by the authors, without undue reservation.

## Ethics Statement

The studies involving human participants were reviewed and approved by Indiana University Institutional Review Board. The ethics committee waived the requirement of written informed consent for participation.

## Author Contributions

JK conceptualized the study, conducted analysis and interpretation of data, and wrote the first draft of the manuscript. LD conceptualized the study, interpreted data, and contributed to writing. EN and OR conducted analysis, contributed to interpretation of data, and contributed to writing. GL and SP conceptualized study, conducted data interpretation, and contributed to writing. All authors contributed to the article and approved the submitted version.

## Conflict of Interest

The authors declare that the research was conducted in the absence of any commercial or financial relationships that could be construed as a potential conflict of interest.
