# Supplementary figures and images for: Adversity History Predicts Self-Reported Autonomic Reactivity and Mental Health in US Residents During the COVID-19 Pandemic
Source: Front Psychiatry. 2020 Oct 27;11:577728. doi: 10.3389/fpsyt.2020.577728 (PMC7653174; doi:10.3389/fpsyt.2020.577728)

**Supplementary Data Sheet 1.** Consort diagram


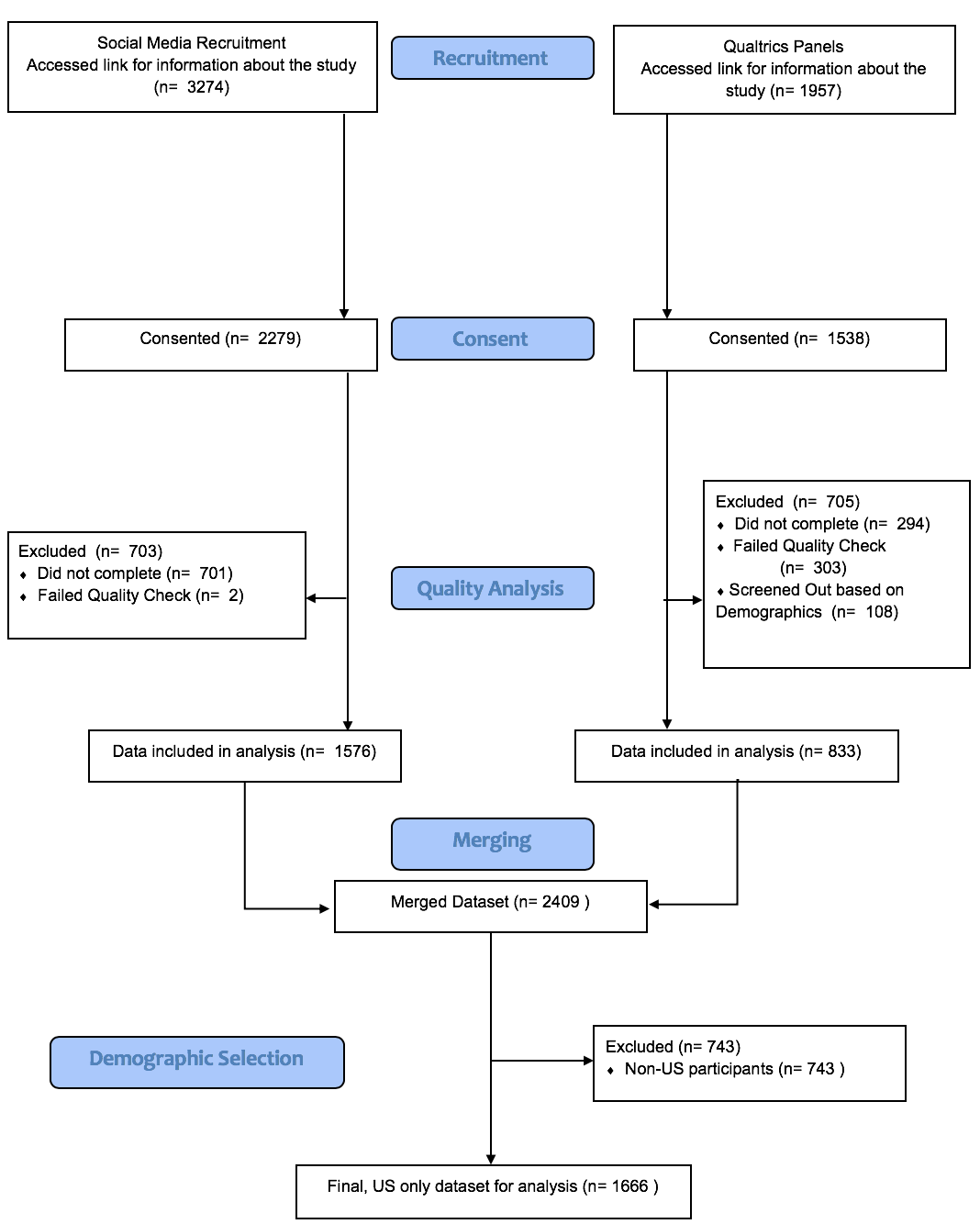

Supplement: Supplementary file 1 [file Data_Sheet_1.docx]
